# Concurrency of Early-Age Exposure to Chinese Famine and Diabetes Increases Recurrence of Ischemic Stroke

**DOI:** 10.3389/fneur.2020.520633

**Published:** 2021-01-20

**Authors:** Yue Suo, Weiqi Chen, Yuesong Pan, Hao Li, Xia Meng, Zixiao Li, Chunjuan Wang, Jing Jing, Yilong Wang, Yongjun Wang

**Affiliations:** ^1^Department of Neurology, Beijing Tiantan Hospital, Capital Medical University, Beijing, China; ^2^China National Clinical Research Center for Neurological Diseases, Beijing, China; ^3^Center of Stroke, Beijing Institute for Brain Disorders, Beijing, China; ^4^Beijing Key Laboratory of Translational Medicine for Cerebrovascular Disease, Beijing, China

**Keywords:** exposure to famine, diabetes, metabolic syndrome, outcome, stroke

## Abstract

**Background and Purpose:** Early age exposure to the Chinese Great Leap Forward famine (1959–1961) is associated with the incidence of risk factors for ischemic stroke. This study aims to examine the relationship between early age famine exposure and 12-month stroke recurrence. We sought to explore the interaction between famine exposure status and metabolic phenotypes on stroke recurrence and how the adherence of crucial evidence-based key performance indicators (KPI) would modify this interaction.

**Methods:** We analyzed data of patients who were born between 1953 and 1964 in the China National Stroke Registry II (CNSR-II). The study population was further divided into five subgroups for comparing 12-month stroke recurrence. A multivariate Cox proportional hazard regression model was used in analyzing the impact of the concurrence of metabolic phenotypes—type 2 diabetes (T2D) or metabolic syndrome (MetS)—and early-age famine exposure on recurrent risk. The influence of the adherence to predefined KPI and concurrency of metabolic phenotype was also evaluated.

**Results:** Concurrent T2D and early age famine exposure was associated with an increased recurrence risk of ischemic stroke with 12 months [adjusted hazard ratio (HR): 1.63, 95% confidence interval (CI) 1.28–2.07]. Optimal adherence to KPI was not associated with significantly reduced risk of 12-month stroke recurrence (adjusted HR: 0.80, 95% CI: 0.51–1.26).

**Conclusions:** Concurrency of early-age famine exposure and diabetes mellitus was associated with a higher risk of stroke recurrence within 12 months, and adherence to evidence-based KPI did not reduce the risk significantly.

## Introduction

Early life famine exposure is one of the critical environmental stress factors that might add risk to non-communicable diseases in adulthood (1–5). According to the Developmental Origins of Health and Disease/fetal programming hypothesis, a series of adaptions beneficial to survival would take place when facing an adverse environment at an early age and alter the structures as well as functions of the individual's organs permanently (6, 7). The Chinese Great Leap Forward (GLF) famine during 1959–1961 caused ~30 million excessive deaths (8). Previous studies indicate the long-term adverse influence of early age famine exposure is related to hypertension, coronary artery disease, hyperglycemia, and metabolic syndrome (MetS) (2, 9–13).

Stroke is the leading contributor to the burden of neurological disorders globally that cause a considerable burden all over the world (14–17). It is so far still unclear how early age famine exposure is involved in the risk of ischemic stroke or diabetes mellitus later in life, or the effect of famine exposure on the association between diabetes and ischemic stroke. Previous studies found a sex-specific association between early age (fetal and childhood) exposure to the Chinese GLF famine and elevated risk of MetS (3, 18, 19). In areas that were severely affected during the famine, early age exposure was associated with a higher prevalence of MetS (20). Relationship between early age exposure to famine and increased risk of type 2 diabetes mellitus (T2D) or hyperglycemia was reported (3, 21). Higher glycosylated hemoglobin (HbA_lc_) was observed in individuals with early age famine exposure (22).

Inconsistent conclusions about the association between early age (pre-natal or post-natal) exposure to famine and the risk of ischemic stroke are reported. In a study using data derived from the Dutch famine cohort, there was no significant effect of pre-natal exposure to famine on the risk of stroke (23). However, a significantly lower risk of stroke in post-natal Dutch famine–exposed women was observed (1).

Evidence from several prospective registries or randomized studies links T2D, pre-diabetic status, or insulin resistance with outcomes of ischemic stroke (24, 25). According to the previous study, concurrent MetS and T2D are risk modifiers of stroke recurrence (26).

Optimal adherence with in-hospital or discharge performance measurements is reported to improve the 12-month outcome after ischemic stroke (27). How adherence to specific performance measurements would interact with famine exposure status and concurrent T2D/MetS on stroke outcomes is yet not discussed.

The current study aimed to analyze the influence of early age exposure to the Chinese GLF famine on ischemic stroke outcomes. We also intended to explore whether famine-exposure status would exacerbate the impact of MetS or T2D on stroke recurrence risk by comparing the long-term (12-month) recurrence rate after ischemic stroke. Furthermore, we aimed to analyze whether adherence to the key performance measurements would improve long-term outcomes among patients with concurrent early age famine exposure and T2D.

## Methods

### Participants and Data Extraction

Data are derived from the second phase of the China National Stroke Registry II (CNSR-II) study. Detailed design and methodology were published previously (28). Briefly, CNSR-II is a prospective, nationwide, hospital-based registry of acute stroke within 7 days of the index cerebrovascular event. From June 2012 to January 2013, participants were recruited consecutively from 219 hospitals enrolled in the Chinese Stroke Center Alliance, including 31 provinces, municipalities, and autonomous regions in mainland China.

Demographic information, clinical data, and neurological evaluation were extracted by screening medical documents by trained research coordinators at each study center (28).

The central institutional ethics committee approved the CNSR-II study protocol from each ethics committee of the participating hospitals before participants' enrollment.

Among all participants of CNSR-II discharged with computed tomography (CT)/magnetic resonance imaging (MRI) confirmed diagnosis of ischemic stroke were included. Because the exact date of the starting and ending of the Chinese famine was unavailable, patients born between 1958 and 1959 and those born between 1961 and 1962 were excluded to minimize the possibility of misclassification. A sensitivity analysis, including people born between 1958 and 1959 and between 1961 and 1962, is conducted.

### Exposure to Famine in Different Period of Life

Individual famine exposure status was evaluated by birth year reported by participants or their legal representatives. Those birth years reported according to the Chinese lunar calendar were converted to Gregorian calendar years.

First, subjects of this study were divided into five groups according to birth year: born in 1953 and 1954, born in 1955 and 1956, born between 1957 and 1958, born between 1959 and 1961, and born between 1962 and 1964 and categorized as late childhood exposed, mid-childhood exposed, early childhood exposed, fetal exposed and non-exposed (2, 29). The non-exposed group was considered to be the control group in subsequent analysis.

Second, all included ischemic stroke patients were divided into two groups: (1) the exposed to famine group: those who were born in the famine exposure period (1953–1958, 1959–1961) and (2) the not exposed to famine group: patients whose birth year is not in the famine exposure period (born after 1962).

### Definition of T2D and MetS

T2D was ascertained by self-report history, receiving hypoglycemic treatment, or fasting plasma glucose ≥126 mg/dL (7.0 mmol/L). MetS was diagnosed according to the Chinese Diabetes Society (CDS) definition, which requires three or more of the following risk factors: (1) BMI ≥ 25; (2) fasting plasma glucose of 110–125 mg/dL (6.1–6.9 mmol/L), 2 h plasma glucose ≥ 140 mg/dL (7.8 mmol/L), or on antidiabetic medication; (3) blood pressure ≥140/≥90 mmHg, history of hypertension or on antihypertensive medication; and (4) serum triglycerides ≥ 150 mg/dL (1.7 mmol/L) or high-density lipoproteins <35 mg/dL (0.9 mmol/L) in men and <39 mg/dL (0.9 mmol/L) in women (30).

### Follow-Up and Outcome Definition

Follow-up data were obtained by trained coordinators using a standard script through telephone interview in each participating hospital at 3, 6, and 12 months after symptom onset of the index event (29).

Recurrent stroke, which includes both ischemic and hemorrhagic stroke, was collected and analyzed during hospitalization, 3, 6, and 12 months after symptom onset of the index event (31). We focused on the 12-month recurrent stroke in the current analysis.

### Key Performance Measurement Index of Stroke Care

The evidence-based performance measurement index is mostly consistent with the GOLDEN BRIDGE-AIS research protocol (31), including four performance measurements at the acute phase of ischemic stroke and five performance measurements at discharge. Except for smoking cessation, the other six measurements are identical to the American Heart Association—Get With The Guideline (AHA-GWTG) measurements (32). We also included stroke education, which would be provided to patients or caregivers as a measurement concerning weight loss, dietary modification, physical activity, and smoking cessation advisement (33).

Optimal compliance with the performance measurement index was defined as eligible patients for each item in the index who received the relevant intervention and received stroke education.

### Statistical Analysis

Baseline characteristics were first tabulated between inclusion and exclusion groups and against the life period of famine exposure to discover potential confounding factors. We summarize the continuous variables as the median with interquartile ranges and present the categorical variables as frequency and percentage. We used the Kruskal–Wallis test to compare skewed continuous variables and ordinal variables. We compared the categorical variables using the χ^2^ statistics or Fisher exact test as appropriate. We used multivariate Cox proportional hazard regression models to calculate hazard ratios (HR) of the risk of stroke recurrence between famine-exposed groups and non-exposed groups. Kaplan–Meier curves were drawn to analyze the cumulative risks of outcomes. We used the multivariable-adjusted model to estimate how the optimal compliance with performance measurements may influence the 12-month recurrence. For analyzing the interaction effect between MetS and famine exposure status as well as between T2D and famine exposure on the risk of stroke recurrence at 12 months, we used multivariate Cox regression models. A two-sided *P* <0.05 is considered to reach statistical significance. The statistical analyses are performed by using SAS version 9.4 software (SAS Institute Inc., Cary, NC, USA).

We intend to perform a causal mediation analysis to evaluate the contribution of T2D or MetS to the association between early-age famine exposure and 12-month stroke recurrence using the “mediation” package in R (R Foundation for Statistical Computing, Vienna, Austria) (34).

## Results

### Patient Characteristics

**Figure 1:**
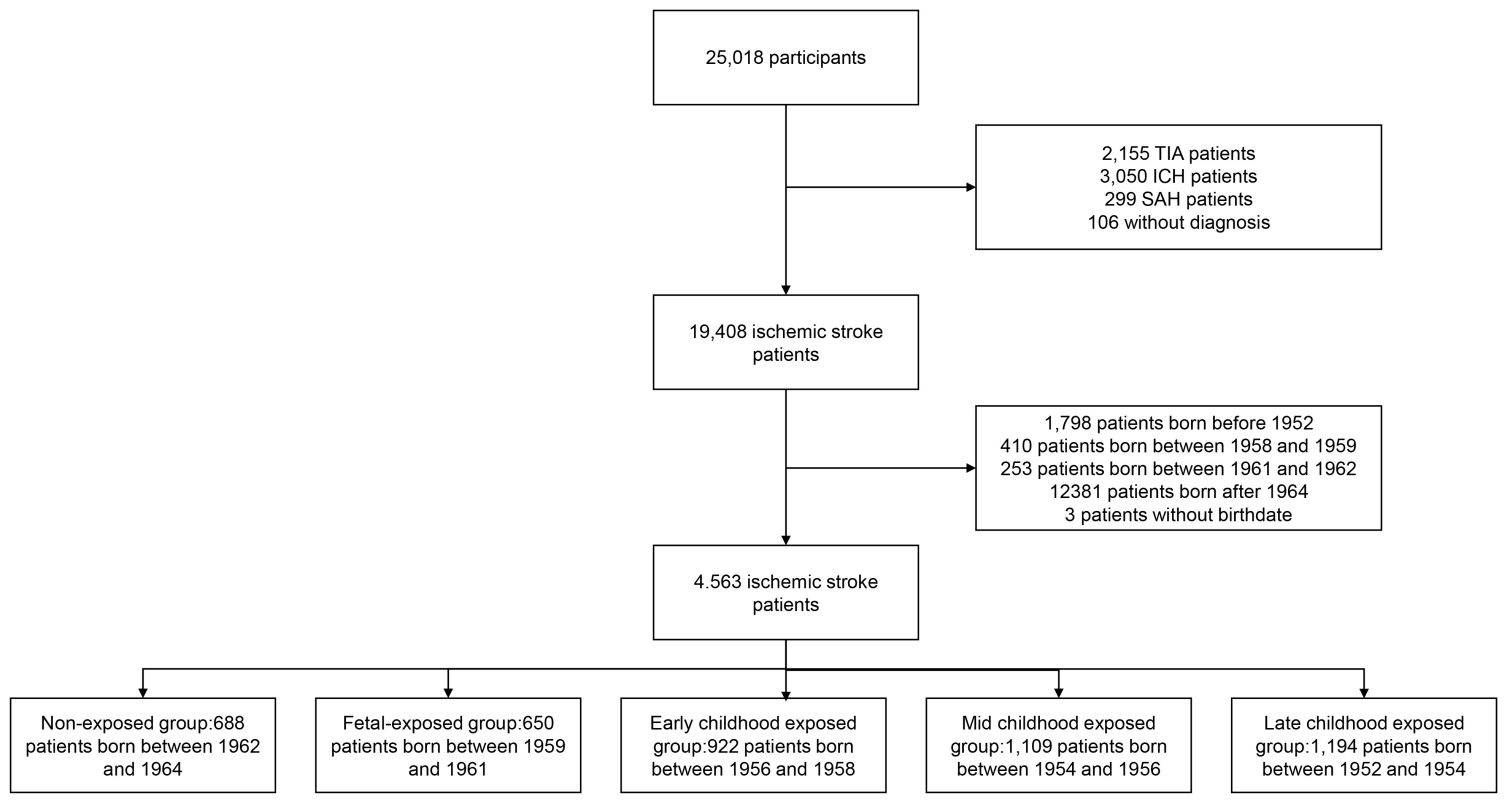
Flow diagram of study participant selection and grouping of included participants. TIA indicates transient ischemic attack. ICH indicates intracerebral hemorrhage. SAH indicates subarachnoid hemorrhage.

The final diagnosis and age at enrollment were available for 24,912 acute stroke patients in the CNSR-II study. Among all 19,408 ischemic stroke patients, we included 45,63 patients, including 3,875 patients who were exposed to the Chinese famine, and 688 non-exposed patients (as reference) for the analysis (Figure 1). Among the included cohort of patients, the mean age was 56 years, and 69.49% were men (Supplementary Table 1).

Based on the birthdate determined by age at enrollment and inclusion date, 688 patients were in a non-exposed subgroup, 650 patients in the fetal-exposed subgroup, 922 patients in the early childhood exposed subgroup, 1,109 patients in the mid-childhood exposed subgroup, and 1,194 patients in the late-childhood exposed subgroup (Figure 1). Most of the baseline characteristics were well-balanced among subgroups (Table 1). We observed more male patients in the fetal-exposed group, fewer patients with a diabetes history in the non-exposed group, and fewer patients with smoking or drinking habits in the late childhood exposure group.

**Table 1:** Baseline characteristics of included participants by different life phase of early age when exposed to the Chinese GLF.

**Characteristics**	**Non-exposed** **(*n* = 688)**	**Fetal exposed** **(*n* = 650)**	**Early-childhood exposed** **(*n* = 922)**	**Mid-childhood exposed** **(*n* = 1,109)**	**Late-childhood exposed** **(*n =* 1,194)**	***P*-value**
Men, *n* (%)	493 (71.66%)	475 (73.08%)	638 (69.2%)	795 (71.69%)	770 (64.49%)	<0.001
mRS prior to current event, median (IQR)	0 (0-1)	0 (0-1)	0 (0-1)	0 (0-1)	0 (0-1)	<0.001
Age (y), median (IQR)	49 (49–50)	52 (52–53)	55 (55–56)	57 (57–58)	60 (59–60)	<0.001
Prior antiplatelet use, *n* (%)	106 (15.41%)	119 (18.31%)	157 (17.03%)	210 (18.94%)	211 (17.67%)	0.39
Medical history, *n* (%)						
Ischemic stroke	170 (24.71%)	185 (28.46%)	252 (27.33%)	319 (28.76%)	339 (28.39%)	0.38
TIA	28 (4.07%)	35 (5.38%)	48 (5.21%)	51 (4.6%)	48 (4.02%)	0.55
Intracerebral hemorrhage	7 (1.02%)	20 (3.08%)	29 (3.15%)	32 (2.89%)	33 (2.76%)	0.07
Subarachnoid hemorrhage	0 (0%)	1 (0.15%)	2 (0.22%)	2 (0.18%)	2 (0.17%)	0.85
Atrial fibrillation	25 (3.63%)	26 (4.00%)	35 (3.80%)	46 (4.15%)	63 (5.28%)	0.37
Diabetes mellitus	110 (15.99%)	155 (23.85%)	196 (21.26%)	220 (19.84%)	265 (22.19%)	<0.01
Hypertension	425 (61.77%)	412 (63.38%)	594 (64.43%)	711 (64.11%)	745 (62.4%)	0.75
Dyslipidemia	80 (11.63%)	89 (13.69%)	115 (12.47%)	146 (13.17%)	142 (11.89%)	0.71
Myocardial infarction	12 (1.74%)	20 (3.08%)	14 (1.52%)	24 (2.16%)	22 (1.84)	0.25
Perivascular disease	20 (2.91%)	26 (4%)	42 (4.56%)	50 (4.51%)	51 (4.27%)	0.48
Dementia	1 (0.15%)	0 (0%)	1 (0.11%)	1 (0.09%)	0 (0%)	0.69
Mental disturbance	3 (0.44%)	3 (0.46%)	2 (0.22%)	3 (0.27%)	2 (0.17%)	0.73
Liver or kidney insufficiency	1 (0.15%)	3 (0.46%)	8 (0.87%)	7 (0.63%)	9 (0.75%)	0.39
Prosthetic heart valve	2 (0.29%)	2 (0.31%)	2 (0.22%)	1 (0.09%)	7 (0.59%)	0.29
Ever smoke	390 (56.69%)	396 (60.92%)	501 (54.33%)	624 (56.27%)	571 (47.82%)	<0.001
Drinking history	309 (44.91%)	287 (44.15%)	349 (37.85%)	424 (38.23%)	381 (31.91%)	<0.001
Laboratory results, median (IQR)						
BUN, mmol/L	4.72 (3.79-5.80)	4.78 (3.91-5.95)	4.84 (4.00-5.87)	4.96 (4.09-6.00)	5.00 (4.19-6.00)	<0.001
CR, umol/L	66 (55.20-77.00)	68 (57.40-79.20)	68 (56.00-80.80)	68 (57.00-81.00)	68 (58.00-81.00)	0.03
CRP, mg/L	3.78 (1.58-6.30)	3.85 (1.41-7.50)	3.72 (1.80-7.10)	4.00 (2.00-6.50)	3.40 (1.56-6.30)	0.79
Fasting glucose, mmol/L	5.46 (4.80-6.48)	5.52 (4.99-7.21)	5.57 (4.88-7.10)	5.50 (4.86-6.70)	5.59 (4.89-7.20)	0.02
HCY, umol/ml	14.70 (10.53-21.60)	15.00 (10.60-22.60)	14.30 (10.60-21.07)	14.66 (10.86-20.15)	15.10 (10.80-21.65)	0.82
LDL-C, mmol/L	2.72 (2.18-3.26)	2.81 (2.22-3.40)	2.84 (2.25-3.44)	2.82 (2.25-3.36)	2.77 (2.24-3.38)	0.07
HDL-C, mmol/L	1.11 (0.93-1.31)	1.08 (0.93-1.28)	1.13 (0.95-1.32)	1.11 (0.95-1.34)	1.13 (0.96-1.36)	0.04
TG, mmol/L	1.51 (1.07-2.17)	1.57 (1.09-2.26)	1.51 (1.04-2.21)	1.49 (1.08-2.18)	1.44 (1.03-2.10)	0.03
BMI	24.22 (22.60-26.42)	24.22 (22.86-26.12)	24.13 (22.49-26.12)	24.14 (22.55-25.82)	24.14 (22.49-25.95)	0.02
Stroke severity, median (IQR)	3 (2-6)	4 (2-6)	4 (2-6)	3 (2-6)	4 (2-7)	0.63

*Mrs, indicates modified Rankin Scale; IQR, indicates interquartile range; TIA, indicates transient ischemic attack; BUN, indicates blood urea nitrogen; CR, indicates creatinine; CRP, indicates C-Reactive Protein; HCY, indicates homocysteine; LDL-C, indicates low density lipoprotein cholesterol; HDL-C, indicates high density lipoprotein cholesterol; TG, indicates triglyceride; BMI, indicates body mass index*.

### Famine Exposure at Different Periods of Life and Stroke Outcome

**Figure 2:**
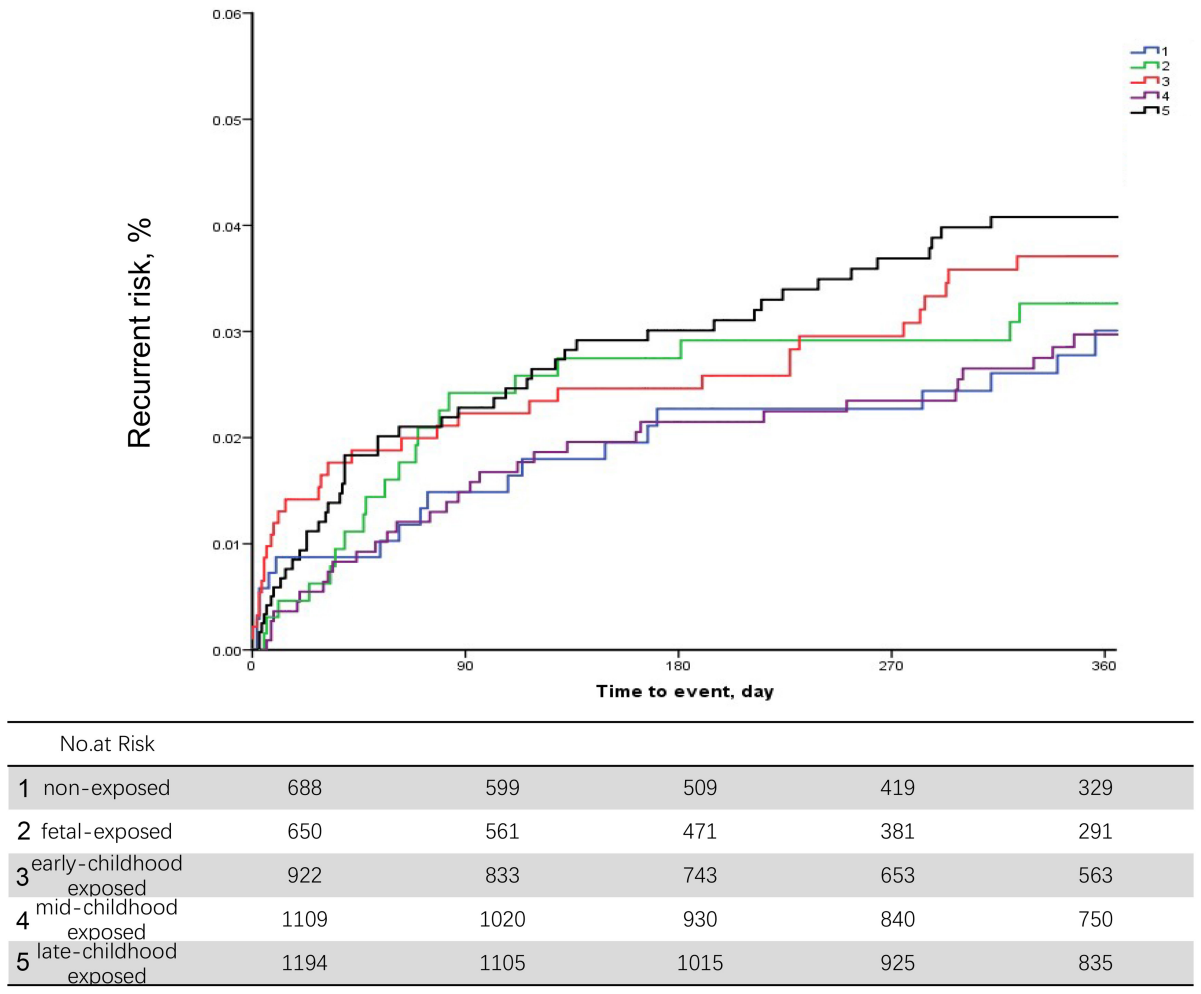
Unadjusted Kaplan–Meier curves for stroke recurrence in non-exposed, fetal exposed, early-childhood exposed, mid-childhood exposed, and late-childhood exposed patients.

During the 12-month follow-up, 227 patients (4.97% of the study population) had a recurrent stroke. After adjusting for age and execution of clinical pathways, we found no significant associations between different early age period famine exposure and recurrent stroke within 12 months (Table 2). Discrimination of long-term recurrence or mortality among different famine exposure categories was not significant in the unadjusted Kaplan–Meier curves (Figure 2).

**Table 2:** Recurrence within 12-month of exposed cohorts compared to non-exposed cohort.

**Endpoints**	**Non-exposed cohort**	**Fetal-exposed cohort**	**Early-childhood exposed cohort**	**Mid-childhood exposed cohort**	**Late-childhood exposed cohort**
Recurrence (%) at 12 months					
*P*^a^		0.52	0.62	0.75	0.83
Hazard ratio (95% CI)^a^	Ref	1.33 (0.56–3.15)	1.46 (0.33–6.49)	1.38 (0.20–9.69)	1.29 (0.12–14.33)
*P*^b^		0.48	0.47	0.59	0.69
Hazard ratio (95% CI)^b^	Ref	1.37 (0.57–3.26)	1.74 (0.39–7.71)	1.71 (0.24–11.96)	1.63 (0.15–18.23)

a Evaluating the overall risk of four exposed cohort with non-exposed as reference adjusted for age, execution of clinical pathways.

b *Evaluating the risk of four exposed cohorts with non-exposed as reference adjusted for age, sex, history of ischemic stroke, myocardial infarction, atrial fibrillation, hypertension, diabetes, dyslipidemia, National Institute of Stroke Scale at admission and modified Rankin Scale before the index stroke*.

When we further adjusted for conventional cerebrovascular risk factors, including medical history of ischemic stroke, myocardial infarction, atrial fibrillation, hypertension, diabetes mellitus, and dyslipidemia as well as NIHSS score at admission and mRS before index stroke, the association between early age famine exposure status and 12-month stroke recurrence was not significant. The results were stable after sensitivity analysis (Supplementary Table 2).

In the multivariate analysis, no significant differences of 12-month recurrence were discovered among different groups of famine exposure age (Table 3).

**Table 3:** Twelve-month recurrence among patients receiving medical care adherence to performance measurements vs. routine care.

**Endpoints**	**Non-exposed cohort**	**Fetal-exposed cohort**	**Early-childhood exposed cohort**	**Mid-childhood exposed cohort**	**Late-childhood exposed cohort**
Recurrence (%) at 12 months					
*P*^a^		0.49	0.59	0.71	0.81
Hazard ratio (95% CI)^a^	Ref	1.36 (0.57-3.23)	1.51 (0.34-6.72)	1.46 (0.21-10.21)	1.35 (0.12-15.08)
*P*^b^		0.48	0.46	0.30	0.65
Hazard ratio (95% CI)^b^	Ref	1.37 (0.57-3.27)	1.75 (0.39-7.77)	1.72 (0.25-12.05)	0.65 (0.15-18.36)

a Evaluating the overall risk of four exposed groups compared to non-exposed group after adjusted for age, sex.

b *Evaluating the risk of four exposed groups with non-exposed group after adjusted for age, sex, history of ischemic stroke, myocardial infarction, atrial fibrillation, hypertension, diabetes, dyslipidemia, National Institute of Stroke Scale at admission, modified Rankin Scale before the index stroke and optimal adherence to key performance index*.

### Association Between MetS, Famine Exposure, and Risk of Stroke Recurrence at 12 Months

A total of 1273 (6.56%) events of stroke recurrence were recorded during a 12-month follow-up. The interaction effect between MetS and famine exposure status was not significant (*P* = 0.15 for interaction) on 12-month recurrent risk in multivariate analysis.

After adjusting for age and gender, patients with MetS, and not exposed to famine had a higher risk of stroke recurrence (adjusted HR 1.36, 95% CI: 1.01–1.67). After adjusting for all the potential confounding factors (gender, age, history of smoking, and history of drinking), patients with MetS and not exposed to famine had a higher risk of stroke recurrence (8.46 vs. 6.73%; adjusted HR 1.23, 95% CI: 1.02–1.46).

### Association Between T2D, Famine Exposure, and Risk of Stroke Recurrence at 12 Months

**Figure 3:**
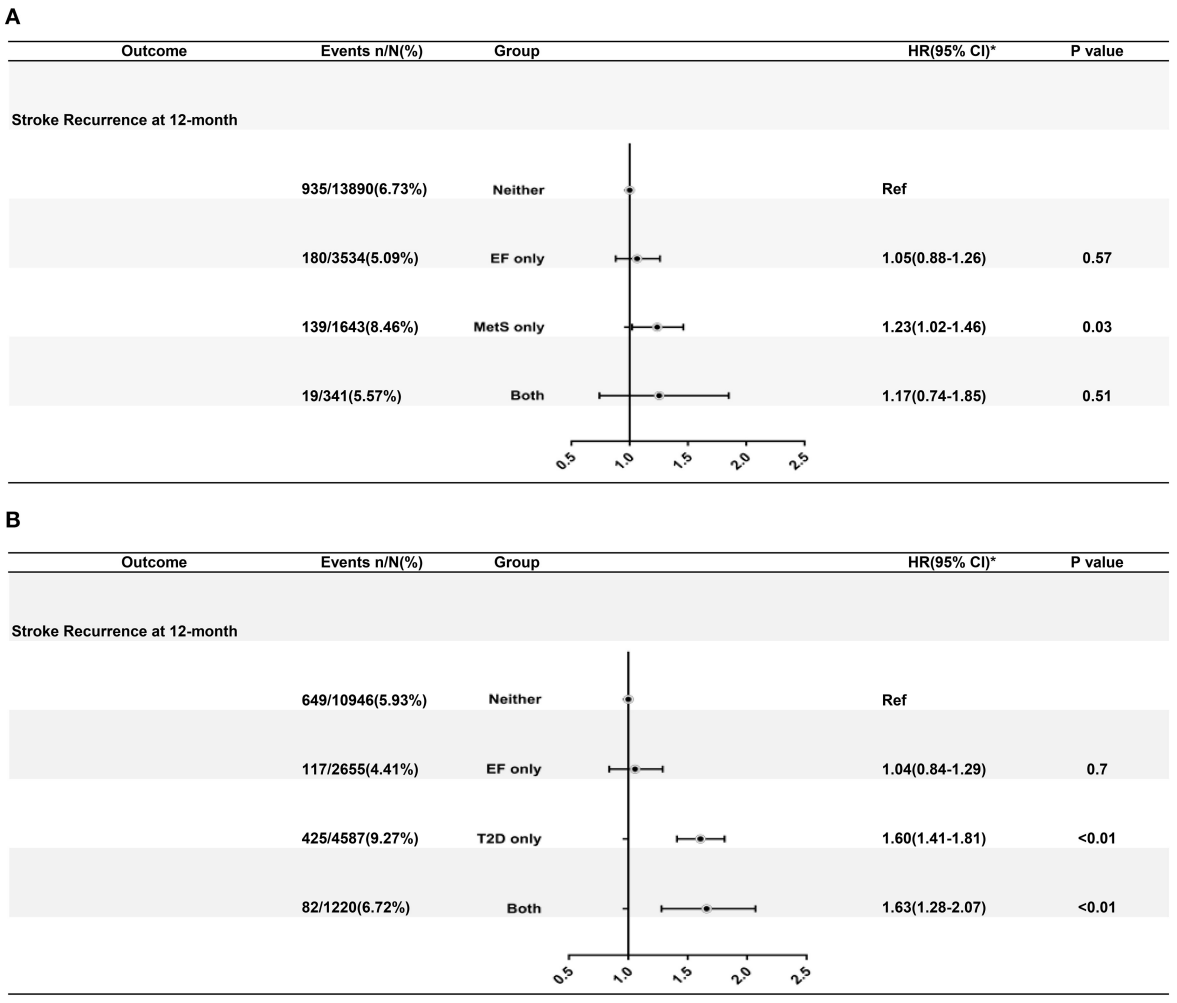
**(A)** Adjusted hazard ratios of 12-month stroke recurrence according to CDS defined MetS and famine exposure status. **(B)** Adjusted hazard ratios of 12-month death from any cause according to T2D and famine exposure status. Estimation of hazard ratios was after adjusting for age, sex, history of ischemic stroke, myocardial infarction, atrial fibrillation, hypertension, diabetes mellitus, dyslipidemia, National Institutes of Health Stroke Scale at admission, and modified Rankin Scale before index stroke. HR indicates hazard ratio, CI indicates confidential interval, EF indicates exposure to famine, MetS indicates metabolic syndrome, T2D indicates type 2 diabetes mellitus.

As shown in Figure 3, a significant interaction effect between T2D and famine exposure was observed (*P* < 0.0001 for interaction) on recurrent stroke risk during a 12-month follow-up. Patients with T2D only (9.27 vs. 5.93%; adjusted HR: 1.60, 95% CI: 1.41–1.81) and T2D with famine exposure (6.72 vs. 5.93%; adjusted HR: 1.63, 95% CI: 1.28–2.07) had increased risk of stroke recurrence at 12 months. Mediation analysis revealed significant effects for T2D on the pathway between early age famine exposure and 12-month stroke recurrence (Supplemental Figure 1).

### Compliance With Performance Measurement and 12-Month Outcomes in Different Status of Famine Exposure With/Without T2D

Optimal compliance with performance measurements was not associated with reduced 12-month stroke recurrence in patients with early age famine exposure and T2D [HR 95% CI = 0.80 (0.51–1.26), *P* = 0.34]. (Supplementary Table 3).

## Discussion

In the nationwide multicenter stroke cohort–CNSR-II study, we analyzed the association between early age exposure to Chinese GLF famine and outcomes of ischemic stroke. Across fetal, early childhood, mid-childhood, and late childhood famine exposure, gradients of changing risks to different results were not discovered. We detected a significant interaction effect between famine exposure status and T2D on 12-month recurrence. Patients with T2D only or both T2D and exposure to famine had an increased risk of stroke recurrence at 12 months. The residual risk of 12-month stroke recurrence was observed despite optimal compliance with KPI in patients with early age famine exposure and T2D.

Compared with the few studies that examined the association of pre-natal exposure to famine and risk of incident stroke, our results about the impact of fetal famine exposure on stroke recurrence were also not reaching statistical significance. In the Dutch famine birth cohort with 1,177 individuals, the pre-natal exposure group was subdivided into three gestation stage exposure subgroups. None of these groups showed different risks of non-fatal stroke compared to the non-exposed subgroup (23).

Studies that focused on post-natal famine exposure reported inconsistent results. The post-natal exposure to starvation was related to a higher risk of cardiovascular disease, including stroke (35). In the longitudinal follow-up of male residents exposed to the siege of Leningrad, mortality due to ischemic stroke was increased in those exposed to the siege at mid to late childhood and puberty (36, 37). In a women-only Dutch famine cohort, early childhood and early adulthood famine exposure were related to a lower risk of stroke (1).

In our study, postnatal famine exposure has not shown an impact on the risk of recurrent stroke. The famine's different duration may cause the discrepancy in results from other studies and our study, men-only/women-only cohort or cohort of both genders, fatal ischemic stroke, and recurrence after index stroke.

Exposure to famine at an early life stage was associated with unhealthy lifestyles, including lack of physical activity and smoking in the female population (38). From our baseline characteristics, patients who were exposed to the Chinese GLF were more likely to have a history of smoking.

The potential mechanism of early age exposure to suboptimal conditions and risk of chronic diseases later in life were postulated by Hales and Baker and referred to as early life programming (39). Insufficiency of a fetal stage or early post-natal stage could be proposed to trigger adaptions made for maintaining vital organ function, such as brain function, at the expense of other organ-development restriction, such as the pancreas, kidney, skeletal muscle, and so on. The adaptational changes could lead to permanent changes in organ structure and be responsible for an elevated risk of chronic disease in adulthood, such as T2D, MetS, cardiovascular disease, and hypertension (40). The hypothesis is supported by islet beta-cell reduction and vascularization in rat maternal protein restriction and maternal caloric restriction models (41–43). Recently, a study focusing on epigenetic reprogramming during the early gestation period acquired DNA methylation data of 24 pre-natal Dutch famine exposure individuals and 24 unexposed siblings as controls using reduced representation bisulfite sequencing. Differential methylation occurred at regulatory regions in famine-exposed individuals, and the areas were involved in pathways regulating metabolic phenotypes, such as birth weight and serum LDL cholesterol levels. This indicates that the influence of early development stage exposure to famine on epigenetic modulation would further harm metabolic capabilities later in adulthood (44).

Current studies do not discuss the interaction between early age famine exposure and MetS or T2D with recurrent stroke to the best of our knowledge. As a frequent comorbidity of stroke, MetS was not related to an increased risk of recurrence of minor or lacunar stroke, but concurrent MetS and T2D add the recurrence risk (45, 46). Consistent and relatively robust correlation between early age exposure to the Chinese GLF and increased female-specific risk of MetS or T2D were discovered (11, 19, 20, 47–49). Our study detects the influence of the interaction between T2D and early-age famine exposure on long-term (12-month) stroke recurrence but no interaction between MetS and famine exposure. Ischemic stroke patients with concurrent T2D and exposure to the Chinese GLF had a higher risk of recurrence through 12 months after the initial index event. Constant risk factor monitoring and management is necessary for these patients. It is also important for these patients to stick to a healthy lifestyle for better prevention of recurrent cerebrovascular disease. According to our results, whether exposed to famine or not, adherence to the evidence-based performance measurement index may improve long-term survival in patients without MetS. Evidence-based interventions are necessary for all ischemic stroke patients, especially in patients without MetS.

Our study has several limitations. First, the national registry from which we derived the data did not collect patients' birthplace, relevant nutritional indicators, and follow-up nutritional status. The severity of the Chinese GLF varied across the whole mainland, measured by excessive mortality, and we were unable to stratify the severity because of the lack of patients' birthplace. Also, we could not include the follow-up nutritional status or relevant nutritional indicators in the research scope. More focused research about nutrition and stroke recurrence are needed. Second, the “nutritional rich” period and fast development in economics were a confounding factor interacting with early age exposure to food shortage. Uneven and different speeds of development among cities and rural areas might introduce bias in our analysis. Third, early age exposure to famine may also cause problematic eating attitudes later in life as an adverse behavioral risk factor for overweight and MetS. Further stratification of famine severity, region of residence, and eating behavior in future studies is needed to validate and interpret the current results. Fourth, there was an inevitable age difference within each exposure group, which could interact with the impact of famine exposure on stroke recurrence. We included age in the adjustment of the multivariate analysis, and the association between early age famine exposure and 12-month stroke recurrence was also not significant.

Along with the economic improvement and reformation in mainland China after the GLF famine, the transition from food shortage to food surplus reshaped the diet toward an unhealthy pattern with a cereal-based, high-fat, high-energy, and limited protein food structure (50). To monitor and prevent nutrition-related non-communicable diseases, dietary guidance might need to be adjusted to keep up with the speed of transformation in economics and living conditions.

## Conclusions

Concurrent T2D and early age famine exposure were associated with an increased recurrence risk of ischemic stroke within 12 months. The residual risk of 12-month stroke recurrence was observed despite optimal compliance with KPI in patients with early age famine exposure and T2D.

## Data Availability Statement

The datasets generated for this study are available on request to the corresponding author.

## Ethics Statement

The studies involving human participants were reviewed and approved by The ethics committee of Beijing Tiantan Hospital. The patients/participants provided their written informed consent to participate in this study.

## Author Contributions

This research was conceptualized by YS and WC. Data collection was done by ZL, CW, JJ, and XM. Statistical analysis was undertaken by YP and HL. YS and WC prepared the manuscript. All authors approved the protocol.

## Conflict of Interest

The authors declare that the research was conducted in the absence of any commercial or financial relationships that could be construed as a potential conflict of interest.
