# Supplementary material for: Concurrency of Early-Age Exposure to Chinese Famine and Diabetes Increases Recurrence of Ischemic Stroke
Source: Front Neurol. 2021 Jan 20;11:520633. doi: 10.3389/fneur.2020.520633 (PMC7855705; doi:10.3389/fneur.2020.520633)
Supplement: Supplementary file 1 [file Data_Sheet_1.docx]

Supplemental Figure 1**.** Mediation analysis of the effect for T2D on the pathway between early-age famine exposure and 12-month stroke recurrence.

**Supplementary Table 1. Baseline characteristics of participants who were diagnosed as ischemic stroke by included in and excluded from the analysis.**

| **Characteristics** | **Included**  **(n=4563)** | **Excluded**  **(n=14845)** | ***P* value** | |
| --- | --- | --- | --- | --- |
| Men, n(%) | 3171(69.49%) | 9137(61.55%) | <0.001 |  |
| mRS prior to current event, median (IQR) | 0(0-1) | 0(0-1) | <0.001 |  |
| Age(y), median (IQR) | 56(53-59) | 69(63-76) | <0.001 |  |
| Prior antiplatelet use, n(%) | 803(17.6%) | 3041(20.49%) | <0.001 |  |
| Medical history, n(%) |  |  |  |  |
| Ischemic stroke | 1265(27.72%) | 4825(32.5%) | <0.001 |  |
| TIA | 210（4.6%） | 592（3.99%） | 0.07 |  |
| Intracerebral hemorrhage | 121(2.65%) | 412(2.78%) | 0.66 |  |
| Subarachnoid hemorrhage | 7(0.15%) | 18(0.12%) | 0.60 |  |
| Atrial fibrillation | 195(4.27%) | 1182(7.96%) | <0.001 |  |
| Diabetes mellitus | 946(20.73%) | 3103(20.90%) | 0.80 |  |
| Hypertension | 2887(63.27%) | 9693(65.29%) | 0.01 |  |
| Hyperlipidemia | 572(12.54%) | 1778(11.98%) | 0.31 |  |
| Myocardial infarction | 92(2.02%) | 385(2.59%) | 0.03 |  |
| Perivascular disease | 189(4.14%) | 607(4.09%) | 0.87 |  |
| Dementia | 3(0.07%) | 89(0.60%) | <0.001 |  |
| Mental disturbance | 13(0.28%) | 36(0.24%) | 0.62 |  |
| Liver or kidney insufficiency | 28(0.61%) | 127(0.66%) | 0.11 |  |
| Prosthetic heart valve | 14(0.31%) | 30(0.20%) | 0.19 |  |
| Sickle cell disease | 0 | 1(0.01%) | 0.58 |  |
| Ever smoke | 2482(54.39%) | 6116(41.20%) | <0.001 |  |
| Drinking history | 1750(38.35%) | 4045(27.25%) | <0.001 |  |
| Laboratory results, median (IQR) |  |  |  |  |
| BUN, mmol/L | 4.90(4.03-5.95) | 5.20(4.20-6.43) | <0.001 |  |
| CR, umol/L | 68(57-80) | 71(59-86) | <0.001 |  |
| CRP, mg/L | 3.80(1.67-6.40) | 4.50(2.00-8.11) | 0.03 |  |
| Fasting glucose, mmol/L | 5.51(4.88-6.96) | 5.50(4.85-6.74) | <0.001 |  |
| HCY, umol/ml | 14.80(10.70-21.40) | 16.00(11.60-22.80) | <0.001 |  |
| LDL-C, mmol/L | 2.80(2.23-3.38) | 2.72(2.18-3.32) | <0.001 |  |
| HDL-C, mmol/L | 1.11(0.94-1.33) | 1.14(0.96-1.37) | <0.001 |  |
| TG, mmol/L | 1.50(1.06-2.19) | 1.30(0.95-1.89) | <0.001 |  |
| BMI | 24.21(22.49-26.03) | 23.92(21.97-25.69) | <0.001 |  |
| Treatment, n(%) |  |  |  |  |

| Antiplatelet | 3798(97.58%) | 11829(96.88%) | 0.02 |
| --- | --- | --- | --- |
| Dual antiplatelet | 308(7.99%) | 725(5.99%) | <0.001 |
| Stroke severity, median(IQR) | 4(2-6) | 4(2-7) | <0.001 |

mRS indicates modified Rankin Scale; IQR indicates interquartile range; TIA indicates transient ischemic attack; BUN indicates blood urea nitrogen; CR indicates creatinine; CRP indicates C-Reactive Protein; HCY indicates homocysteine; LDL-C indicates low density lipoprotein cholesterol; HDL-C indicates high density lipoprotein cholesterol; TG indicates triglyceride; BMI indicates body mass index.

**Supplementary Table 2. Recurrence of exposed cohorts compared to non-exposed cohort.**

| **Endpoints** | **Non-exposed cohort** | **Patients born between 1961 and 1962** | **Fetal-exposed cohort** | **Patients born between 1958 and 1959** | **Early-childhood exposed cohort** | **Mid-childhood exposed cohort** | **Late-childhood exposed cohort** |
| --- | --- | --- | --- | --- | --- | --- | --- |
| Recurrence (%) at 12 months |  |  |  |  |  |  |  |
| *P*^a^ |  | 0.87 | 0.42 | 0.16 | 0.39 | 0.50 | 0.59 |
| Hazard ratio (95% CI)^a^ | Ref | 0.93(0.39-2.20) | 1.43(0.60-3.38) | 2.33(0.72-7.56) | 1.92(0.44-8.33) | 1.95(0.29-13.30) | 1.93(0.18-20.85) |

a:Evaluating the risk of four exposed cohorts with non-exposed as reference adjusted for age, sex, history of ischemic stroke, myocardial infarction, atrial fibrillation, hypertension, diabetes, dyslipidemia, National Institute of Stroke Scale at admission and modified Rankin Scale before the index stroke.

**Supplementary Table 3. Multivariate analysis of factors associated with 12-month recurrence in patients with early-age famine exposure and T2D.**

|  | **12-month recurrence** | |
| --- | --- | --- |
|  | **HR (95%CI)** | **P** |
| Age(y), median (IQR) | 0.99(0.91-1.08) | 0.90 |
| Men, n(%) | 0.93(0.58-1.50) | 0.77 |
| Medical history |  |  |
| Ischemic stroke | 1.49(0.94-2.37) | 0.09 |
| Myocardial infarction | 0.86(0.21-3.52) | 0.83 |
| Atrial fibrillation | 1.83(0.73-4.57) | 0.19 |
| Diabetes | 0.83(0.51-1.33) | 0.43 |
| Dyslipidemia | 1.26(0.74-2.15) | 0.40 |
| Baseline NIHSS | 1.04(1.01-1.08) | 0.01 |
| mRS before index stroke | 1.27(1.08-1.48) | <0.01 |
| Adherence to key performance index | 0.80(0.51-1.26) | 0.34 |

HR indicates hazard ratio; CI, confidence interval; NIHSS, mRS indicates modified Rankin Scale and IQR indicates interquartile range.
