# Supplementary figures and images for: Concurrency of Early-Age Exposure to Chinese Famine and Diabetes Increases Recurrence of Ischemic Stroke
Source: Front Neurol. 2021 Jan 20;11:520633. doi: 10.3389/fneur.2020.520633 (PMC7855705; doi:10.3389/fneur.2020.520633)

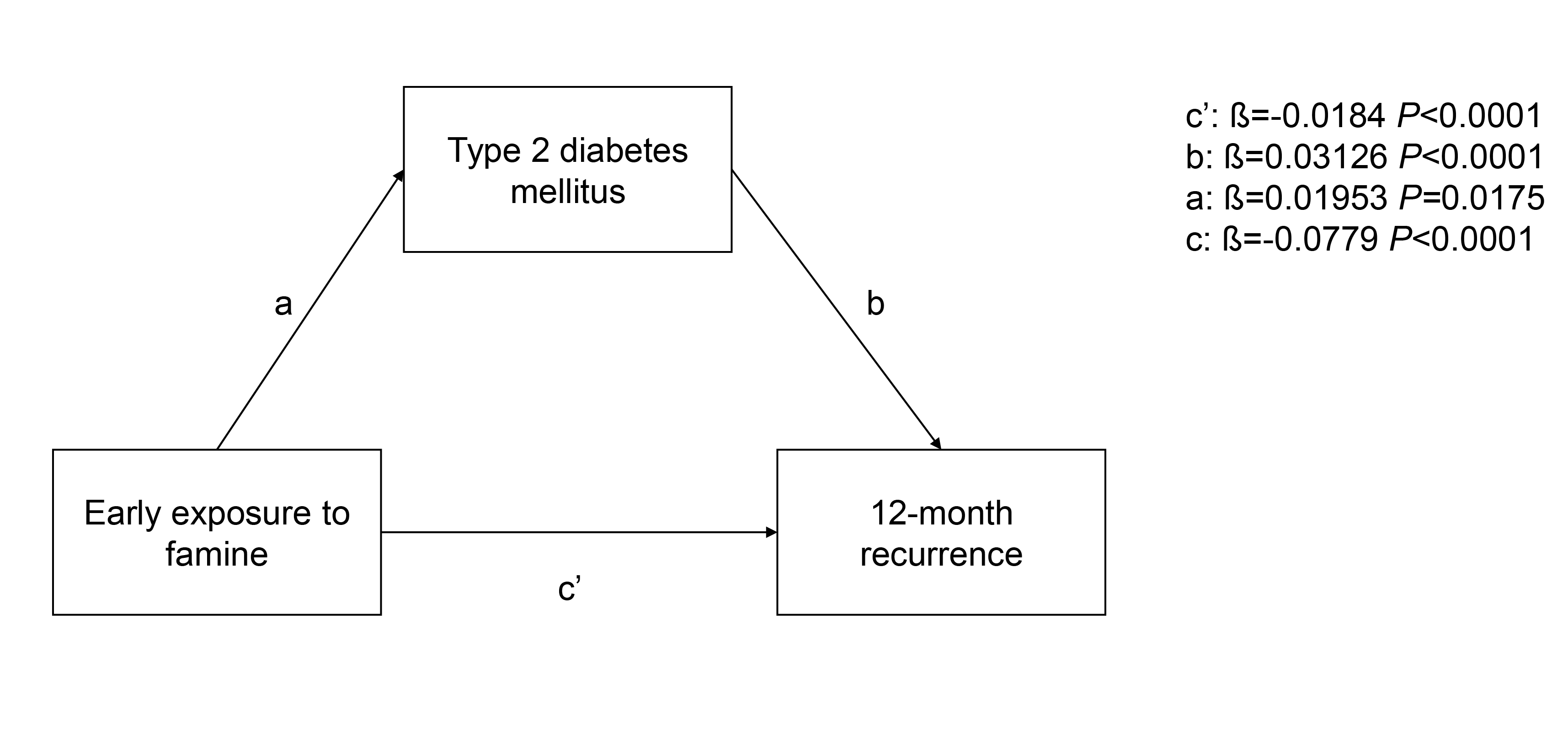

Supplement: Supplementary file 2 [file Image_1.TIF]
